# Satiation and Stress-Induced Hypophagia: Examining the Role of Hindbrain Neurons Expressing Prolactin-Releasing Peptide or Glucagon-Like Peptide 1

**DOI:** 10.3389/fnins.2012.00199

**Published:** 2013-01-21

**Authors:** James W. Maniscalco, Alison D. Kreisler, Linda Rinaman

**Affiliations:** ^1^Department of Neuroscience, University of PittsburghPittsburgh, PA, USA

**Keywords:** noradrenergic, HPA axis, food intake, rats, mice

## Abstract

Neural circuits distributed within the brainstem, hypothalamus, and limbic forebrain interact to control food intake and energy balance under normal day-to-day conditions, and in response to stressful conditions under which homeostasis is threatened. Experimental studies using rats and mice have generated a voluminous literature regarding the functional organization of circuits that inhibit food intake in response to satiety signals, and in response to stress. Although the central neural bases of satiation and stress-induced hypophagia often are studied and discussed as if they were distinct, we propose that both behavioral states are generated, at least in part, by recruitment of two separate but intermingled groups of caudal hindbrain neurons. One group comprises a subpopulation of noradrenergic (NA) neurons within the caudal nucleus of the solitary tract (cNST; A2 cell group) that is immunopositive for prolactin-releasing peptide (PrRP). The second group comprises non-adrenergic neurons within the cNST and nearby reticular formation that synthesize glucagon-like peptide 1 (GLP-1). Axonal projections from PrRP and GLP-1 neurons target distributed brainstem and forebrain regions that shape behavioral, autonomic, and endocrine responses to actual or anticipated homeostatic challenge, including the challenge of food intake. Evidence reviewed in this article supports the view that hindbrain PrRP and GLP-1 neurons contribute importantly to satiation and stress-induced hypophagia by modulating the activity of caudal brainstem circuits that control food intake. Hindbrain PrRP and GLP-1 neurons also engage hypothalamic and limbic forebrain networks that drive parallel behavioral and endocrine functions related to food intake and homeostatic challenge, and modulate conditioned and motivational aspects of food intake.

## Introduction

Factors that increase or decrease food intake do so by altering meal size, meal frequency, or both (Smith, 1998, 1999, 2000). Satiation – the natural process that ends a meal – is a brainstem-mediated phenomenon in which food intake is terminated as a consequence of intake within that meal, thus influencing meal size. In contrast, satiety is a post-ingestive state that precludes initiation of a meal, thereby influencing meal frequency. Satiation occurs in adult decerebrate rats in which the brainstem is surgically isolated from the hypothalamus and the rest of the forebrain (Grill and Norgren, 1978; Seeley et al., 1994; Grill and Kaplan, 2002; Grill, 2010), and in neonatal rats with functionally immature forebrain-brainstem connections (Hall and Bryan, 1980; Hall and Swithers-Mulvey, 1992; Rinaman et al., 1994). Satiation depends on peripherally generated “satiety signals,” which decrease activity in brainstem circuits that maintain ingestive licking/chewing/swallowing behaviors, and/or increase activity in brainstem circuits that suppress these behaviors (Smith, 1998, 1999, 2000). Although the brainstem is sufficient for satiation, the amount of food consumed before a meal is voluntarily terminated is powerfully modulated by neural signals from the forebrain and hormonal factors that increase or decrease the behavioral potency of satiety signals (Grill and Hayes, 2012). Some of these signals and factors act directly on the brainstem components of ingestive control circuits, while others act indirectly by engaging hypothalamic and limbic forebrain regions that influence the activity of brainstem ingestive control circuits via descending projections (Smith, 1998, 1999, 2000; Grill and Kaplan, 2002; Luckman and Lawrence, 2003; Grill and Hayes, 2009; Grill, 2010). Satiety signals and other feedback about the quality and quantity of food consumed are delivered to brainstem and forebrain regions that coordinate a host of feeding-related processes, including anticipatory and reflexive metabolic, endocrine, and autonomic adjustments, preference and avoidance learning, appetitive motivation, and behavioral state control (Smith, 1999; Grill and Kaplan, 2002; Luckman and Lawrence, 2003; Grill and Hayes, 2009, 2012; Grill, 2010; Rinaman, 2010; Berthoud et al., 2011).

Satiation and satiety are normal, everyday processes. However, consuming a satiating meal can be stressful, especially if food intake occurs at an unusual (i.e., unpredicted) time, or if the meal is unusually large or calorically dense. Food intake presents an immediate physiological challenge to homeostasis (Woods, 1991), and there is evidence that feeding activates the neuroendocrine hypothalamic-pituitary-adrenal (HPA) axis to increase circulating levels of glucocorticoids (i.e., cortisol in humans, corticosterone in rats and mice; Shiraishi et al., 1984; Dallman et al., 2004). The HPA axis is primarily involved in energy storage and mobilization under baseline conditions and in response to homeostatic challenge (Dallman et al., 2004), and stressors are commonly defined as internal or external stimuli that increase HPA axis activity above baseline circadian-modulated levels. Meals are fundamentally disruptive to homeostasis because they cause significant changes in a variety of important physiological parameters that are under constant surveillance and regulation, such as gastrointestinal distension, liver temperature, osmotic pressure, and blood glucose (Woods, 1991; Woods and Ramsay, 2000). Viewed from this perspective, it is reasonable to propose that there is a very fine line between central circuits that inhibit food intake during satiation and satiety, and those that inhibit intake during acute stress. The idea that satiety signals and hypophagic stressors might recruit a common set of neurons and circuits is not new (e.g., see Ritter et al., 1999; Seeley et al., 2000; Calvez et al., 2011), but the putatively shared circuits whose recruitment results in decreased meal size and/or frequency remain to be identified.

The present review discusses evidence that inhibition of food intake by satiety signals and by hypophagic stressors is mediated, at least in part, by recruitment of two phenotypically distinct but anatomically intermingled populations of hindbrain neurons. The first population comprises noradrenergic (NA) neurons within the caudal nucleus of the solitary tract (cNST; A2 cell group), a majority of which express prolactin-releasing peptide (PrRP; Maruyama et al., 2001). PrRP was identified as an endogenous ligand for the human orphan G-protein-coupled receptor hGR3/GPR10, and earned its name because it induces prolactin secretion from anterior pituitary cells *in vitro* (Hinuma et al., 1998). However, PrRP is absent from the external layer of the median eminence, and there is no evidence that endogenous PrRP plays any physiological role in prolactin release. Instead, mRNA for PrRP receptor (hGR3/GPR10) is expressed in multiple brainstem and forebrain regions implicated in feeding, behavioral, and physiological responses to stress (Roland et al., 1999; Lawrence et al., 2000; Yamada et al., 2009). PrRP mRNA is expressed exclusively by a subset of caudal medullary NA neurons, and by a small number of neurons in a ventral region of the caudal dorsomedial hypothalamic nucleus (Iijima et al., 1999; Roland et al., 1999; Onaka et al., 2010). The second group of hindbrain neurons with a proposed role in both satiation and stress-induced hypophagia synthesize glucagon-like peptide 1 (GLP-1). Despite the largely overlapping hindbrain distribution of PrRP and GLP-1 neurons, the latter are a completely distinct population of non-adrenergic neurons that expresses mRNA for preproglucagon (PPG), the protein precursor of GLP-1. Within the brain, PPG mRNA expression is limited to the olfactory bulb, the cNST, and the caudal medullary reticular formation (Larsen et al., 1997; Merchenthaler et al., 1999)^1^. Since PPG-expressing neurons within the olfactory bulb are interneurons with very short axons, GLP-1 fibers, and terminals throughout the rest of the CNS can be assumed to originate from hindbrain PPG-expressing neurons.

Results from many published reports indicate that food intake in rats and mice is reduced after central infusions of PrRP, GLP-1, or their synthetic analogs (Tang-Christensen et al., 1996; Turton et al., 1996; Imeryüz et al., 1997; McMahon and Wellman, 1997, 1998; Asarian et al., 1998; Thiele et al., 1998; Lawrence et al., 2000, 2002, 2004; Kinzig et al., 2002; Schick et al., 2003; Grabauskas et al., 2004; Bechtold and Luckman, 2006; Nakade et al., 2006; Takayanagi et al., 2008; Holmes et al., 2009; Takayanagi and Onaka, 2010; Hayes et al., 2011; Alhadeff et al., 2012). Such studies are important, and provide a strong foundation for the hypothesis that both neural populations drive hypophagia. However, delivery of synthetic peptides or their analogs into the brain is a poor model for understanding whether stimulus-induced release of endogenous PrRP or GLP-1 contributes to satiation or stress-induced hypophagia. The present review focuses on results from a smaller number of studies providing evidence that satiety signals and acute stress inhibit food intake by recruiting endogenous PrRP and GLP-1 signaling pathways. Before reviewing those data, we first review the anatomical location, neurochemical features, and circuit connections of hindbrain PrRP and GLP-1 neurons.

## Anatomy of the Dorsal Vagal Complex and Its Resident PrRP and GLP-1 Neurons

**Figure 1:**
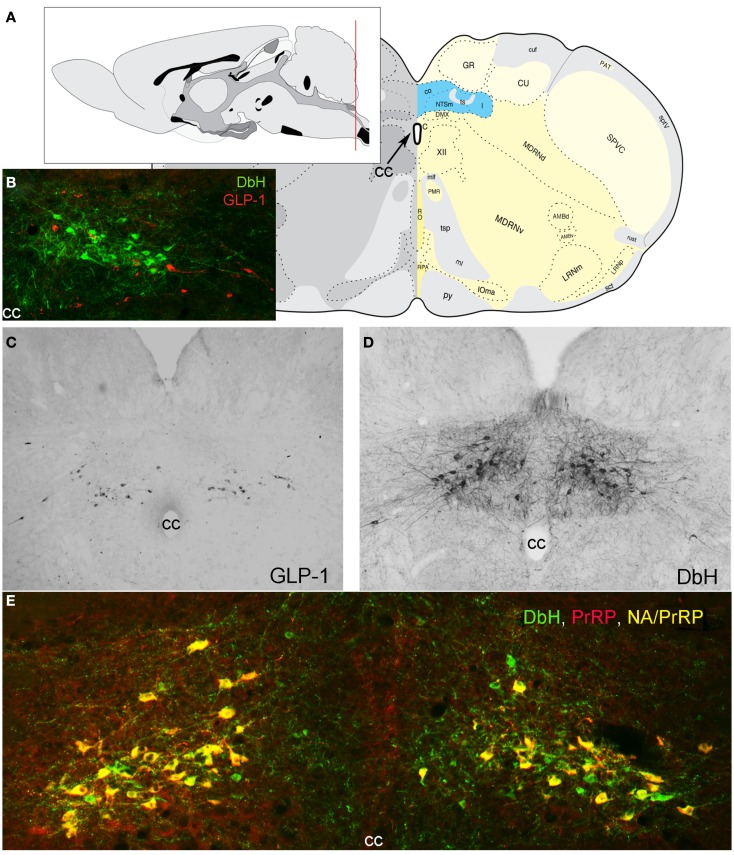
**Location of PrRP and GLP-1 neurons in the rat hindbrain**. **(A)** Schematics illustrating the location of the cNST (highlighted in blue), adapted from Swanson (2004). The red line in the mid-sagittal brain schematic at upper left illustrates the rostrocaudal level of all coronal sections depicted in images. **(B)** In this image, dopamine beta hydroxylase (DbH) immunopositive NA neurons are green, while GLP-1-immunopositive neurons are red. The two intermingled populations are distinct, with no colocalization of immunolabeling. **(C)** GLP-1 immunoperoxidase-labeled neurons. **(D)** DbH immunoperoxidase-positive NA neurons of the A2 cell group. **(E)** In this image, all PrRP-positive neurons are double-labeled for DbH, rendering them yellow/orange (NA/PrRP neurons). Some intermingled NA neurons (green) are PrRP-negative, *cc, central canal*.

Prolactin-releasing peptide-immunopositive neurons and non-adrenergic GLP-1-immunopositive neurons are co-distributed in the hindbrain near the medullary-spinal junction, within caudal levels of the NST and the nearby medullary reticular formation (Figure 1). The cNST is the “visceral” NST, distinct from the more rostral “gustatory” NST (Lundy and Norgren, 2004). The cNST is a key component of the dorsal vagal complex (DVC), which also includes the area postrema (AP) and dorsal motor nucleus of the vagus (DMV). The DVC is remarkable for being perhaps the smallest circumscribed brain region whose destruction is incompatible with life. It is a critical central node for autonomic and endocrine functions, relaying interoceptive visceral, hormonal, and somatic feedback from body to brain, tuning stress responsiveness, and regulating glucose homeostasis and other aspects of energy balance (Zagon et al., 1999; Rinaman, 2003b, 2007, 2010, 2011; Berthoud et al., 2006; Grill and Hayes, 2009, 2012; Grill, 2010; Zhang et al., 2010). The AP and a significant portion of the subjacent cNST contain fenestrated capillaries, allowing blood-borne factors to affect neurons in this region (Yamamoto et al., 2003). As recently reviewed (Grill and Hayes, 2009; Grill, 2010; Rinaman, 2010, 2011), AP neurons innervate the subjacent cNST (Shapiro and Miselis, 1985a; Kachidian and Pickel, 1993; Cunningham et al., 1994), and cNST neurons innervate other NST neurons (including those located in the more rostral “taste” area) as well as gastrointestinal and pancreatic vagal preganglionic parasympathetic motor neurons whose cell bodies occupy the DMV and whose dendrites ramify widely within the overlying cNST (Shapiro and Miselis, 1985b).

In addition to inputs from the AP, cNST neurons receive sensory feedback from cardiovascular, respiratory, and alimentary systems (Kalia and Sullivan, 1982). Visceral sensory inputs arrive predominantly via glutamatergic glossopharyngeal and vagal afferents whose central axons converge in the solitary tract before synapsing with the dendrites and somata of cNST neurons, including GLP-1 and NA neurons, the latter of which undoubtedly includes the PrRP-positive majority subpopulation (Altschuler et al., 1989; Rinaman et al., 1989; Spyer, 1990; Bailey et al., 2006; Appleyard et al., 2007; Hisadome et al., 2010, 2011). In the mouse *in vitro* slice preparation, glutamatergic visceral afferent signals produce tightly synced, large-amplitude excitatory postsynaptic currents in NA and GLP-1 neurons within the cNST, providing high-fidelity transmission of sensory nerve activity. Other visceral and somatic sensory inputs are relayed to the cNST from the spinal cord, trigeminal and related nuclei, and reticular formation (Menétrey and Basbaum, 1987; Arbab et al., 1988; Altschuler et al., 1989; Menétrey and De Pommery, 1991; de Sousa Buck et al., 2001).

Given the diversity of sensory signals they are positioned to receive, it is not surprising that GLP-1 and NA neurons, including PrRP neurons, respond to a broad array of interoceptive signals that can suppress food intake and also drive the HPA axis, including hormonal, thermal, osmotic, gastrointestinal, cardiovascular, respiratory, and inflammatory signals (Sawchenko and Swanson, 1981; Luckman, 1992; Rinaman et al., 1993, 1997, 1998; Chan and Sawchenko, 1994, 1998; Dayas et al., 2001a; Hollis et al., 2004; Rinaman, 2004, 2010; Myers and Rinaman, 2005; Myers et al., 2005; Duale et al., 2007; Gaykema et al., 2007; Bienkowski and Rinaman, 2008; Kasparov and Teschemacher, 2008; Takayanagi et al., 2008; Bonnet et al., 2009). In these cited studies and many others, stimulus-induced “activation” of NA, PrRP, and/or GLP-1 neurons generally is characterized by double immunolabeling to localize nuclear cFos, the protein product of the immediate-early response gene, *cfos*, together with cytoplasmic proteins identifying the chemical phenotype of activated neurons. As a binary index of neural activation, the presence or absence of cFos immunolabeling does not index the magnitude or duration of a neuron’s presynaptic inputs, or its resulting spike frequency. However, quantitative and regional analyses of neural cFos expression permit assessment of stimulus-induced activation across multiple populations of phenotypically identified neurons under control and experimental conditions, making it an ideal approach for testing hypotheses regarding neural sensitivity and/or function. The collective results of studies utilizing cFos indicate that NA, PrRP, and GLP-1 neurons are consistently activated by stimuli that present actual or anticipated threats to bodily homeostasis (see below, Potential Role of PrRP and GLP-1 Neurons in Stress-Induced Hypophagia).

Prolactin-releasing peptide and GLP-1 neurons participate in reciprocal connections with the medullary reticular formation, including the caudal ventrolateral medulla (cVLM), and other regions of the pons, diencephalon, and telencephalon that are implicated in food intake and body energy balance (Rinaman, 2010; Grill and Hayes, 2012). Direct descending projections from the cortex, limbic forebrain, and hypothalamus to cNST regions where PrRP and GLP-1 neurons reside provide a route through which emotional and cognitive events can modulate visceral and ingestive responses to diverse threats and opportunities to which the organism is exposed, including conditioned responses that are based on past experience (Sawchenko, 1983; Li et al., 1996; Li and Sawchenko, 1998; Woods and Ramsay, 2000; Dayas and Day, 2001; Taché et al., 2001; Buller et al., 2003; Dayas et al., 2004; Price, 2005; Blevins and Baskin, 2010). In turn, ascending projections from GLP-1 and NA neurons, including PrRP neurons, provide a route through which interoceptive feedback from the gastrointestinal tract and other organ systems can shape hypothalamic and limbic forebrain functions (Sawchenko, 1983; Loewy, 1990; Onaka et al., 1995, 2010; Blessing, 1997; Rinaman and Schwartz, 2004).

### Beyond the cNST

Many reports cited in the present review leave open the possibility that functions ascribed to central signaling by PrRP and/or GLP-1 neurons include signaling from neurons located not within the cNST, but within the nearby medullary reticular formation. PrRP-positive neurons comprise a subset of the cVLM A1 NA cell group (Chen et al., 1999), whereas GLP-1 neurons are scattered in regions somewhat dorsal and medial to the A1 cell group (Vrang et al., 2007; Vrang and Grove, 2011). However, limited evidence suggests that PrRP and GLP-1 neurons within the cNST are functionally distinct from those located within the reticular formation. For example, NA and GLP-1 neurons within the cNST receive direct visceral sensory input (Appleyard et al., 2007; Hisadome et al., 2011), whereas those in the medullary reticular formation do not. This may explain why A2 NA neurons within the cNST are recruited to express cFos in meal-entrained rats that consume a large scheduled meal, whereas cVLM A1 neurons are not activated (Rinaman et al., 1998). PrRP neurons within the cNST also are activated in mice after a single cycle of 24-h food deprivation followed by re-feeding, whereas PrRP neurons in the reticular formation are not (Takayanagi et al., 2008). In addition, the ability of hypoglycemia to increase food intake apparently is mediated by NA neurons within the VLM, and not by neurons within the cNST A2 cell group (Li et al., 2009). There is no published evidence that GLP-1 neurons within the cNST vs. reticular formation project to different brain areas or maintain separate functions, although this possibility should be examined. It’s relevant to note here that non-NA projections from the cNST to the cVLM (Hermes et al., 2006) allow visceral signals to recruit neurons of the A1 cell group (Tucker et al., 1987; Yamashita et al., 1989; Kawano and Masuko, 1996; Bailey et al., 2006; Hermes et al., 2006), and the axons of many A1 neurons (including PrRP-positive neurons) join the ventral NA ascending bundle along with the axons of cNST neurons that project rostrally from the hindbrain (Sawchenko and Swanson, 1981, 1982; Chan et al., 1995). In the absence of specific evidence to discriminate between PrRP or GLP-1 neurons within the cNST vs. medullary reticular formation, a conservative approach dictates that projections and functions ascribed to chemically distinct neurons in either region should be considered likely to be shared by neurons in the other region.

### Other neurochemical features of PrRP and GLP-1 neurons

Prolactin-releasing peptide neurons are phenotypically distinguished by mRNA expression and positive immunolabeling for PrRP as well as tyrosine hydroxylase (TH), the rate-limiting enzyme for dopamine synthesis, together with dopamine beta hydroxylase (DbH), the enzyme that converts dopamine to norepinephrine (NE; Armstrong et al., 1982; see Figure 1 for colocalization of PrRP and DbH immunolabeling within the cNST). A1 and A2 neurons do not express phenylethanolamine *N*-methyltransferase, the enzyme that converts NE to epinephrine and identifies adrenergic neurons of the C1, C2, and C3 cell groups (Dahlström and Fuxe, 1964), which do not express PrRP (Morales et al., 2000). When considering the functional role of PrRP neurons and their axonal projections, it’s important to keep in mind that these neurons release additional signaling molecules from their axon terminals and varicosities. In rats, at least 80% of A2 neurons express mRNA for a homolog of the vesicular glutamate transporter-2 (Stornetta et al., 2002), suggesting that the majority (perhaps all) of PrRP neurons release glutamate along with NE and PrRP from their axon terminals. In addition, subpopulations of catecholaminergic NST neurons are immunopositive for neuropeptide Y (Sawchenko et al., 1985; Everitt and Hökfelt, 1989), nesfatin-1 (Bonnet et al., 2009), dynorphin (Ceccatelli et al., 1992), neurotensin (Riche et al., 1990), and/or pituitary adenylate cyclase-activating polypeptide (Das et al., 2007). The extent to which cNST PrRP neurons co-express these additional signaling molecules remains unclear.

After posttranslational processing by the prohormone convertases PC1/3 and PC2, PPG-expressing neurons generate GLP-1 and several additional peptides for which GLP-1 neurons are immunopositive, including GLP-2, glicentin, intervening peptide-2, and oxyntomodulin (Schafer et al., 1993; Baggio and Drucker, 2007; Vrang and Larsen, 2010). Indirect evidence suggests that beyond PPG-encoded peptides, GLP-1 neurons also are immunopositive for met-enkephalin, somatostatin, and inhibin-β (Sawchenko et al., 1988, 1990; Sawchenko and Pfeiffer, 1995). Apparently, none of these signaling molecules are expressed by cNST PrRP neurons (because none are expressed by NA neurons), and none of the neuropeptides that potentially are co-expressed by PrRP neurons have been localized to GLP-1 neurons. Another notable difference exists between PrRP and GLP-1 neurons in their expression of leptin receptors (Hay-Schmidt et al., 2001). Evidence for direct neuronal sensitivity to leptin has only been presented by one study, in which leptin directly depolarized identified GLP-1 neurons in brainstem slice preparations from transgenic mice (Hisadome et al., 2010). In mice, leptin receptor mRNA is expressed by GLP-1 neurons but not by NA or PrRP-positive cNST neurons (Garfield et al., 2012). Conversely, in rats, NA (and PrRP) neurons express leptin receptor immunolabeling (Ellacott et al., 2002) and exhibit pSTAT3 induction after ip leptin administration (Huo et al., 2008), evidence for direct leptin sensitivity. Rat GLP-1 neurons do not exhibit pSTAT3 induction after ip leptin (Huo et al., 2008), but it is not known whether rat GLP-1 neurons express leptin receptors. Thus, not only do PrRP and GLP-1 neurons appear to display differential leptin sensitivity, their sensitivity appears to be reversed between rats and mice.

### Brainstem and forebrain targets of PrRP and GLP-1 neurons

Prolactin-releasing peptide and GLP-1 neurons are well-positioned to participate in vago-vagal reflexes that modulate gastrointestinal motility, pancreatic hormone release, and other digestive-related autonomic processes associated with satiation and stress-induced hypophagia. GLP-1 and NA neurons, including PrRP neurons, project locally within the DVC and medullary reticular formation, and also to the spinal cord, comprising a subset of pre-autonomic hindbrain neurons implicated in autonomic control of cardiovascular and digestive functions (Fukuda et al., 1987; Rogers et al., 2003; Martinez-Peña-Y-Valenzuela et al., 2004; Hermann et al., 2005; Travagli et al., 2006; Duale et al., 2007; Pearson et al., 2007; Llewellyn-Smith et al., 2011, 2012). Pancreatic and gastric vagal motor neurons express GLP-1R in rats (Wan et al., 2007; Holmes et al., 2009), and the ability of restraint stress to impact intestinal motility is blocked by central GLP-1R antagonism (Gülpinar et al., 2000). Intra-DVC or fourth ventricular microinjection of PrRP or GLP-1 has pronounced effects on vagally mediated gastric motility, and results from *in vitro* slice preparations suggest that PrRP regulates gastric motor function by modulating the efficacy of excitatory synaptic inputs to vagal motor neurons (Grabauskas et al., 2004).

**Figure 2:**
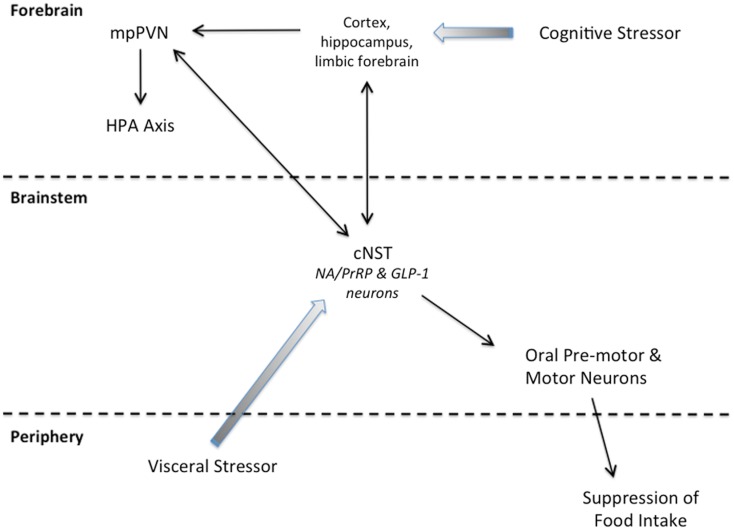
**Summary schematic of our working hypothesis**. Cognitive stressors originate from conditioned and unconditioned cues that are processed through the cortex, hippocampus, and/or limbic forebrain before engaging the HPA axis and cNST. Visceral stressors typically are unconditioned stimuli that first engage cNST neurons (including PrRP and GLP-1 neurons) that innervate hypothalamic and limbic forebrain targets to recruit the HPA axis. Neurons within the cNST engage brainstem targets to organize ingestive motor output.

Axons and varicosities arising from PrRP and GLP-1 neurons also occupy regions of the spinal cord and pontine and medullary reticular formation that contain the pattern generators, pre-motor neurons, and motor neurons that control ingestive consummatory behaviors (i.e., licking/chewing/swallowing; Norgren, 1978; Travers et al., 1997; Chen et al., 2001; Yano et al., 2001; Grill and Kaplan, 2002; Travers and Rinaman, 2002; Chen and Travers, 2003; Grill, 2010). Thus, PrRP and/or GLP-1 neurons may control the behavioral output of ingestive circuits to thereby induce or shape satiation and stress-induced hypophagia (Figure 2). Additional support comes from a transneuronal viral tracing study demonstrating that cNST neurons provide synaptic input to oral pre-motor or motor neurons (Travers and Rinaman, 2002) with demonstrated importance for feeding control (Travers et al., 1997, 2010). It remains to be determined whether PrRP and GLP-1 neurons are among the cNST neurons that are synaptically linked to ingestive pattern generators and oral motor output circuits.

Dual-labeling retrograde tracing and fiber lesion studies indicate that NA neurons, including PrRP-positive neurons, and GLP-1 neurons also project to multiple higher brain regions implicated in behavioral and physiological components of food intake (Morales et al., 2000; Renner et al., 2010, 2012; Rinaman, 2010, 2011). PrRP- and GLP-1-positive fibers have been localized to every medullary, pontine, mesencephalic, diencephalic, and limbic forebrain region that receives axonal input from the cNST. Subsets of A2 neurons, presumably including PrRP neurons, have axon collaterals that innervate two or more forebrain targets (Petrov et al., 1993; Banihashemi and Rinaman, 2006; Schiltz and Sawchenko, 2007; Bienkowski and Rinaman, 2008). One study reported that 11–20% of hindbrain GLP-1 neurons were retrogradely labeled after either PVN or DMH tracer injections, but relatively few (i.e., 15–25%) of the tracer-labeled neurons projected to both hypothalamic nuclei (Vrang et al., 2007). On the other hand, 30–40% of all hindbrain GLP-1 neurons reportedly innervate the midbrain ventral tegmental area (VTA) or the ventral striatal nucleus accumbens (NAcc; Alhadeff et al., 2012), indicating that subsets of GLP-1 neurons probably send collateralized axonal projections to multiple brain regions. Some individual A2 neurons have axons that collateralize to regions of the medullary reticular formation as well as to the limbic forebrain (Reyes and Bockstaele, 2006). Interestingly, however, individual A2 neurons appear to target either the pons or the VLM, but not both (Hermes et al., 2006), suggesting a higher degree of anatomical specificity for projections within the brainstem vs. projections to the hypothalamus and limbic forebrain.

Regarding hypothalamic projections, of particular relevance to the present review is evidence that PrRP and GLP-1 axonal projections target the medial parvocellular subregion of the paraventricular nucleus of the hypothalamus (mpPVN), where they form synaptic contacts with corticotropin releasing hormone (CRH)-positive neurons (Liposits et al., 1986; Matsumoto et al., 2000; Sarkar et al., 2003) at the apex of the HPA axis. CRH is the principal and obligate hypophysiotropic peptide driving the HPA axis under basal conditions and in response to homeostatic challenge (Plotsky et al., 1989; Watts, 1996), and PrRP acts synergistically with NE to activate CRH neurons and the HPA axis (Maruyama et al., 2001; Seal et al., 2002; Uchida et al., 2010). Lesions that decrease NA input to the mpPVN markedly attenuate CRH neuronal cFos activation responses to interoceptive signals (Li et al., 1996; Fraley and Ritter, 2003; Rinaman, 2003a; Ritter et al., 2003; Rinaman and Dzmura, 2007; Schiltz and Sawchenko, 2007; Bienkowski and Rinaman, 2008). Central administration of PrRP or GLP-1 activates cFos in the large majority of CRH-positive mpPVN neurons, and also increases plasma levels of corticosterone (Turton et al., 1996; Rowland et al., 1997; Kinzig et al., 2003; Mera et al., 2006). Although these cFos results by themselves do not prove that CRH neurons are activated directly by PrRP, NE, or GLP-1 receptor-mediated stimulation, bath application of GLP-1 to mouse hypothalamic slices increase spike frequency in a majority of PVN neurons (Acuna-Goycolea and Pol, 2004). Evidence for a mediating role of endogenously released GLP-1 comes from experiments demonstrating that the ability of stress to activate the HPA axis is markedly attenuated in rats after third ventricular administration of a GLP-1 receptor (GLP-1R) antagonist (Kinzig et al., 2003). Interestingly, however, GLP-1R−/− mice display paradoxically *increased* plasma corticosterone levels in responses to acute stress (MacLusky et al., 2000), suggesting that one role of endogenous GLP-1R signaling (at least in mice) may be to restrain or limit stress hormone secretion. This is consistent with the idea that GLP-1 signaling may serve a protective role to limit stress responses in the face of threat, e.g., to limit fever after immune challenge (Rinaman and Comer, 2000), and perhaps to guard against the overconsumption of unanticipated and/or excessively large meals.

As discussed in the Section “Introduction,” the HPA axis is activated by real or perceived homeostatic threats, including *ad libitum* (ad lib) or deprivation-induced food intake. By virtue of their synaptic inputs to CRH neurons, we hypothesize that PrRP and GLP-1 neurons may drive HPA axis responses to food intake, because PrRP and GLP-1 neurons drive HPA axis responses to some (but not all) experimental stressors (Rinaman, 2010, 2011)^2^. In the mature, intact brain, PrRP and GLP-1 neurons innervate not only brainstem but also hypothalamic and limbic forebrain regions that control parallel autonomic, endocrine, and behavioral aspects of satiation and stress-induced hypophagia. In this regard, we view HPA axis activation as occurring in parallel with, but relatively independent from, the brainstem-mediated behavioral processes of satiation and hypophagia, which do not depend on neural connections between the brainstem and forebrain (Grill and Norgren, 1978; Hall and Bryan, 1980; Hall and Swithers-Mulvey, 1992; Rinaman et al., 1994; Seeley et al., 1994; Grill and Kaplan, 2002). The following Section “Potential Role of PrRP and GLP-1 Neurons in Satiation” reviews evidence that PrRP and GLP-1 signaling pathways participate in satiation, and the final Section “Potential Role of PrRP and GLP-1 Neurons in Stress-Induced Hypophagia” reviews evidence that similar signaling pathways contribute to stress-induced hypophagia.

## Potential Role of PrRP and GLP-1 Neurons in Satiation

To examine whether endogenous NA and/or GLP-1 signaling pathways contribute to normal feeding-induced satiation, a few studies have examined whether phenotypically identified NA or GLP-1 neurons within the cNST, the former presumably including PrRP-positive neurons, are differentially activated to express cFos in rats that have recently consumed a large satiating meal, a smaller non-satiating meal, or no meal (Rinaman et al., 1998; Rinaman, 1999b; Gaykema et al., 2009; Kreisler and Rinaman, 2012). In other studies, pharmacological and genetic manipulations have been used to investigate whether rats or mice consume more food when the central receptor-mediated effects of endogenously released PrRP or GLP-1 are attenuated or eliminated (e.g., Schick et al., 2003; Takayanagi et al., 2008; Hayes et al., 2009; Barrera et al., 2011; Dossat et al., 2011; Alhadeff et al., 2012; Dickson et al., 2012). These general experimental approaches are among the best currently available for testing hypotheses about the endogenous central neural underpinnings of satiation and/or stress-induced hypophagia. However, the results of such studies must be interpreted within their own unique experimental context, with particular attention paid to the feeding paradigm utilized and any requisite surgical manipulations and handling.

### Feeding-induced activation of PrRP and GLP-1 neurons

In the absence of experimental manipulations, the cNST displays very low levels of neuronal cFos expression in ad lib-fed (presumably satiated) adult rats that are killed during the first few hours of the light cycle. Under such conditions, identified PrRP and NA neurons rarely express cFos in rats or mice (Takayanagi et al., 2008; Maniscalco and Rinaman, 2013). Conversely, in alternate tissue sections from the same satiated rats, approximately 20% of identified GLP-1 neurons express cFos (Maniscalco et al., 2012). This moderate level of “baseline” GLP-1 activation is markedly reduced (i.e., from ∼20% to less than 4%) if rats are not allowed to eat for 16–24 h before sacrifice (Kreisler and Rinaman, 2012; Maniscalco and Rinaman, 2013). Insofar as neuronal sensitivity is revealed by cFos labeling, these findings suggest that GLP-1 neurons are more sensitive than PrRP neurons to signals associated with post-prandial satiety in non-manipulated, ad lib-fed rats. Post-prandial satiety signals, which may be distinct from signals that promote satiation within a meal, could include gastrointestinal/colonic distension, post-absorptive nutrient levels, and circulating factors such as GLP-1, leptin, insulin, or ghrelin, for which plasma levels differ significantly in satiated rats and mice compared to levels measured after food deprivation (Mizuno et al., 1999; Kmiec et al., 2005, 2006; Johansson et al., 2008).

Under ad lib feeding conditions, laboratory rats typically maintain their body weight by consuming a large number (e.g., 10–15) of small meals each day, with ∼80% of daily food intake occurring nocturnally, and the largest meals predictably consumed near the beginning and end of the dark phase of the photoperiod (Kissileff, 1970; Strubbe et al., 1986; Collier and Johnson, 1990; Woods, 2002). With few exceptions, published studies examining central cFos responses to feeding-induced signals use paradigms in which rats are acclimated over the course of several days or longer to a repeating schedule of food deprivation followed by re-feeding, in order to train them to voluntarily consume a meal that is unusually large compared to the typical size of an ad lib meal. Over the course of a week or two, through a Pavlovian process of classical conditioning, the animal learns to anticipate how much food can be safely consumed during the re-feeding period (including the approximate caloric and macronutrient composition of the meal) as it learns to initiate appropriately timed cephalic phase responses (e.g., increased insulin release, gastric relaxation, digestive enzyme secretion) to ensure that larger meals can be safely consumed (Woods and Ramsay, 2000; Woods, 2002). As pointed out in the Section “Introduction,” these anticipatory adjustments are vitally important, because the energetic benefits of eating are counterbalanced by the energetic cost of homeostatic challenge (Woods, 1991, 2002). Progressive meal-induced recruitment of visceral sensory “satiety” signals from the gastrointestinal tract to the hindbrain serve to constrain meal size during both ad lib and scheduled meal feeding, thereby limiting the stressful effects of food intake (Smith, 1998; Woods, 2002) through a process termed “meal tolerance” (Woods and Ramsay, 2000).

When experimentally naïve rats are food deprived for 24 h and then re-fed for the very first time on chow or palatable liquid diet early in the dark cycle, both NA and GLP-1 neuronal populations are activated to express cFos in direct proportion to the gastric distension produced by the meal (Kreisler and Rinaman, 2012), suggesting that both PrRP and GLP-1 neurons might contribute to meal-induced satiation in this experimental context. Although the PrRP-positive subpopulation of NA neurons has not yet been examined for feeding-induced activation in rats, first time re-feeding after a 24-h fast does activate PrRP neurons in experimentally naïve mice (Takayanagi et al., 2008). After several days of acclimation to a repeating schedule of overnight food deprivation followed by a predictable solid or liquid morning meal, rats voluntarily consume an even larger amount, and cNST NA neurons, presumably including PrRP neurons, still are acutely activated in proportion to the gastric distension produced by the meal (Rinaman et al., 1998). However, in the same rats, GLP-1 neurons are not activated (Rinaman, 1999b). Considered together, these findings suggest that NA and PrRP neurons contribute to meal-induced satiation in both experimental contexts, whereas GLP-1 neurons adapt or acclimate to signals such as gastric distension, elevated blood glucose, or insulin secretion that are predictably generated by a large scheduled meal. Indeed, the hypothesized acclimation of GLP-1 neurons during scheduled meal feeding may be part of the Pavlovian process through which meal-entrained rats learn to tolerate the stress of consuming larger meals. In other words, a lack of GLP-1 neuronal recruitment may represent attenuation or removal of a “brake” on intake that would otherwise constrain meal size via engagement of GLP-1 receptors in the caudal brainstem, which reduces meal size (Hayes et al., 2008, 2009; Grill and Hayes, 2009). If so, then GLP-1 neural recruitment by food intake in rats that are food deprived and then re-fed for the very first time may help explain why these rats consume a smaller meal compared to acclimated, meal-entrained rats.

### Increased food intake after pharmacological blockade of endogenous PrRP and GLP-1 signaling

There currently are no available pharmacological tools with which to antagonize PrRP (hGR3/GPR10) receptors. However, central administration of a monoclonal anti-PrRP antibody in rats was reported to increase meal size but not meal frequency, and to increase total food intake compared to the effects of a control antibody (Takayanagi et al., 2008). These results support the hypothesis that endogenous PrRP signaling participates in meal-induced satiation, but it is unclear where in the brain the proposed signaling occurs or whether satiation can be attributed to hindbrain populations of PrRP neurons as opposed to those located in the dorsomedial hypothalamus. It also is unclear whether meal size and total food intake measured in control rats after central injection of control antibody was reduced compared to similar measures in non-manipulated rats. The importance of including non-manipulated controls is discussed further, below (see the end of this section).

Central GLP-1 signaling can be effectively disrupted by central administration of Exendin-9 (Ex-9), a specific GLP-1R antagonist. Daily intraventricular administration of Ex-9 produces daily increases in food intake compared to intake by rats after vehicle administration (Barrera et al., 2011), although it’s not clear whether this effect depends on increased meal size (supporting a role in satiation), meal frequency (supporting a role in appetite/motivation), or both. Parenchymal administration of Ex-9 into subregions of the mesolimbic reward system, i.e., the VTA or NAcc, increases short-term intake of chow, palatable high-fat diet, and sucrose in rats (Dossat et al., 2011; Alhadeff et al., 2012; Dickson et al., 2012), suggesting that GLP-1 signaling in these regions may normally act to suppress reward-driven intake. Ex-9 targeted to the lateral hypothalamus enhances short-term food intake in ad lib-fed rats, but has no effect on food intake in 24-h food deprived rats (Schick et al., 2003), perhaps because intake in deprived rats already is quite high. Conversely, Ex-9 injections targeted to the fourth ventricle or cNST increase the amount of food consumed by rats after gastric distension, but not after intestinal nutrient infusions (Hayes et al., 2009). Results from these studies support the view that endogenous GLP-1 signaling suppresses or limits food intake across a variety of experimental conditions, and the cNST/hindbrain may be an especially sensitive site of action for this effect (Grill and Hayes, 2012). Indeed, the hypothalamus and forebrain are not required for the ability of GLP-1 signaling to suppress gastric emptying and food intake in rats, as these responses are preserved in chronic supracollicular decerebrate rats (Hayes et al., 2008).

A potential interpretational problem in the studies cited above is the typical comparison of data from surgically manipulated and/or drug-infused rats with data from control rats subjected to sham surgery and/or infused with vehicle. While these are appropriate experimental controls, they are incomplete. The manipulations employed in these studies are often complex, requiring one or more surgical sessions (e.g., to equip animals with chronic brain cannulas or intravenous catheters), and acute handling for central or systemic drug injection. Such manipulations are themselves likely to promote some degree of stress-induced hypophagia, such that “baseline” food intake measured in animals after central or systemic vehicle treatment may be less than intake that would be observed under non-manipulated conditions. Accordingly, the ability of centrally administered Ex-9 or anti-PrRP antibody to increase food intake could be interpreted as evidence that central GLP-1 or PrRP signaling attenuates stress-induced hypophagia. Some experiments have attempted to address this issue by pre-exposing animals to experimental handling and drug infusion conditions in order to habituate them to the potentially stressful aspects of those conditions. However, results in “habituated” animals rarely are compared to results obtained in non-manipulated animals, making it unclear whether or how the habituation procedure affected results. It will be important for future studies to include additional comparative data from non-manipulated controls.

### Increased food intake after genetic manipulation of PrRP and GLP-1 signaling

To better understand the role of endogenous GLP-1 signaling in satiation and long-term energy balance, one research group used a knockdown strategy in which short hairpin RNA was microinjected into the cNTS of adult rats to suppress endogenous PPG expression; this produced a significant and long-lasting increase in daily food intake and body weight compared to control rats (Barrera et al., 2011). However, it might be argued that the brainstem surgery itself in that study had a marked and long-lasting effect to reduce food intake and body weight growth, and that knockdown of PPG expression merely attenuated the deleterious effects of surgery. Evidence challenging a physiological role for GLP-1 signaling in daily food intake control comes from research using GLP-1R−/− mice, which are lean and consume a similar number of daily calories compared to wild-type mice (Scrocchi et al., 1996). In considering this apparent discrepancy in results, a recent review (Vrang and Larsen, 2010) pointed out that GLP-1R−/− mice display an apparent disturbance in satiation, such that termination of food intake is delayed early in the dark period, thereby prolonging the initial nocturnal meal. This is followed by a later suppression of intake to achieve caloric compensation (see Figure 5A in Scrocchi et al., 2000).

GPR10 (PrRP receptor)-deficient mice display hyperphagia under ad lib feeding conditions, but not in a one-time 16 h fasting/re-feeding protocol (Gu et al., 2004). In addition, GPR10 is required for the ability of exogenously administered PrRP and cholecystokinin octapeptide (CCK) to inhibit food intake in mice (Bechtold and Luckman, 2006). PrRP-deficient mice also display hyperphagia and increased body weight when maintained either on normal chow or on a high-fat diet (Mochiduki et al., 2010). In another study, PrRP-deficient mice displayed increased meal size (but not frequency) under ad lib feeding conditions, increased intake after deprivation, and reduced responsiveness to the feeding-suppressive effects of exogenous CCK and leptin (Takayanagi et al., 2008), which endogenously function as satiety signals. Interestingly, a polymorphism in the GPR10 gene that abolishes binding of PrRP in brain slices does not affect the ability of exogenously administered PrRP to suppress food intake in rats (Ellacott et al., 2002), suggesting that the hypophagic effects of the endogenous peptide could also be mediated through another, as yet unidentified, receptor signaling mechanism in rats. Additional studies will be required to examine this issue. However, there is compelling evidence that a natural mutation of the GPR10 receptor (in addition to mutation of CCK-1 receptors) in the Otsuka Long-Evans Tokushima Fatty (OLETF) rat underlies its obese phenotype, and OLETF rats are insensitive to the hypophagic effects of exogenously administered PrRP (Watanabe et al., 2005).

## Potential Role of PrRP and GLP-1 Neurons in Stress-Induced Hypophagia

To the extent that it has been examined, GLP-1, PrRP, and NA neurons within the cNST express cFos in every experimental situation in which food intake is acutely inhibited and the HPA axis is activated (Bouton and Bolles, 1980; Callahan and Rinaman, 1998; Rinaman et al., 1998; Rinaman, 1999b, 2003a, 2004, 2010; Vrang et al., 2003; Zhu and Onaka, 2003; Onaka, 2004; Mera et al., 2006; Gaykema et al., 2007, 2008, 2009; Bonnet et al., 2009; Jelsing et al., 2009; Uchoa et al., 2009). As if to emphasize the close relationship between brainstem neural recruitment and endocrine responses to hypophagic stressors, systemically administered amylin reduces meal size but does not activate NA or GLP-1 neurons, and amylin does not activate the HPA axis (meaning it is not stressful; Potes and Lutz, 2010). Acute stressors that activate cNST NA and GLP-1 neurons, inhibit food intake, and activate the HPA axis also inhibit gastric emptying, likely via direct or indirect NA-, PrRP-, and GLP-1-mediated effects on autonomic outflow (Callahan and Rinaman, 1998; Hellstrom and Naslund, 2001; Rinaman, 2003a, 2004; Rogers et al., 2003; Grabauskas et al., 2004; Nakade et al., 2006; Balcita-Pedicino and Rinaman, 2007; Seto et al., 2008; Hayes et al., 2009).

States of threatened homeostasis are met by a complex but generally predictable repertoire of physiological and behavioral stress responses (Chrousos, 1998; Kyrou and Tsigos, 2009), including suppression of food intake (Dess and Vanderweele, 1994; Calvez et al., 2011). Stress responses are adaptive in the short-term, because they shift the allocation of behavioral and physiological resources away from procuring and storing energy, and toward mobilizing energy and altering behavior to cope with the homeostatic threat. Experimental stressors are diverse in nature and magnitude, but can be categorized as either visceral (a.k.a. interoceptive/physiological), or cognitive (a.k.a. neurogenic/psychological). Visceral stressors typically comprise unconditioned stimuli that present a direct challenge to physiological homeostasis, such as dehydration, toxemia, infection, or gastrointestinal stimulation. Their ability to activate the HPA axis largely depends on direct and relayed projections from spinal and hindbrain viscerosensory neurons to CRH neurons within the mpPVN, including projections from PrRP and GLP-1 neurons (Figure 2). Conversely, cognitive stressors originate from conditioned and unconditioned cues that are processed through the cortex and hippocampus before engaging the limbic forebrain and hypothalamus (Figure 2). Cognitive stressors in rats and mice include predator cues, open illuminated spaces, restraint/immobilization, and conditioned stimuli previously associated with an interoceptive or cognitive stressor. Thus, cognitive stressors predict an impending challenge to homeostasis, including the challenge of a large meal, which can be a visceral stressor. At least some cognitive stressors (i.e., mild footshock and restraint) do not require hindbrain inputs to the mpPVN in order to activate the HPA axis. However, GLP-1, PrRP, and NA neurons are activated by these and other cognitive stressors (Li et al., 1996; Morales and Sawchenko, 2003; Zhu and Onaka, 2003; Maniscalco et al., 2012), likely due to recruitment of descending inputs to the cNST that arise from stress-sensitive regions of the hypothalamus and limbic forebrain (Dayas and Day, 2001; Buller et al., 2003; Dayas et al., 2004; Blevins and Baskin, 2010; Figure 2). Our working hypothesis is that, similar to satiation, stress-induced hypophagia depends on the recruitment of PrRP and GLP-1 neurons that participate in stressor-induced decreases in meal size (Morley et al., 1985). We propose that PrRP and GLP-1 neurons participate in satiation and stress-induced hypophagia regardless of whether these neurons are recruited directly via interoceptive/viscerosensory inputs to the cNST, or indirectly via descending projections from the hypothalamus and limbic forebrain (Figure 2). However, the potential role of PrRP or GLP-1 neurons in mediating stress-induced hypophagia has thus far been examined in only a small number of experimental models. The following paragraphs highlight these relatively limited findings, which cumulatively support the view that the ability of stressors to recruit PrRP and/or GLP-1 neurons contributes importantly to their ability to suppress food intake.

### Cholecystokinin octapeptide

Cholecystokinin octapeptide was the first peptide hormone proposed to act as a physiological within-meal satiety signal (Gibbs et al., 1973). Endogenous CCK is released from the upper intestine by nutrient stimulation during and after a meal, binding to peripheral CCK receptors to thereby increase the activity of glutamatergic vagal sensory inputs to the cNST (Bednar et al., 1994). Without challenging the role of endogenous CCK as a satiety factor, synthetic CCK also has been used as a pharmacological tool to activate central neural circuits that respond to gastric vagal stimulation. Such studies have demonstrated that systemic CCK dose-dependently decreases meal size (West et al., 1984) and elicits cFos activation of cNST neurons (Zittel et al., 1999), including NA neurons (Maniscalco and Rinaman, 2013) that presumably co-express PrRP. Moderate to high doses of CCK (i.e., 10–100 μg/kg BW) activate GLP-1 and NA neurons, including PrRP neurons (Luckman, 1992; Rinaman et al., 1993, 1995; Verbalis et al., 1995; Lawrence et al., 2002; Bechtold and Luckman, 2006; Babic et al., 2009; Maniscalco and Rinaman, 2013) that project to the PVN (Rinaman et al., 1995) and activate CRH and oxytocin neurons (Verbalis et al., 1991). Systemic CCK at doses of ∼1–3 μg/kg BW, which many researchers would argue are within the physiological range, are “stressful” in that they elevate plasma levels of adrenocorticotropic hormone (ACTH) in rats (Kamilaris et al., 1992); higher CCK doses produce larger HPA axis responses. CCK delivered at a lower dose (i.e., 0.5 μg/kg BW) does not activate the HPA axis (Kamilaris et al., 1992). Although hypophagic effects of lower doses of CCK (i.e., ≤0.5 μg/kg BW) have been reported using various systemic routes of administration and dietary conditions, to our knowledge there are no reports of parallel HPA axis activation under these conditions. As the hypothalamus and the entire forebrain are unnecessary for the ability of CCK to inhibit intake (Grill and Smith, 1988), it follows that HPA axis activation also is unnecessary for CCK-induced hypophagia. However, in the absence of evidence indicating otherwise, exogenous CCK-induced hypophagia appears to be accompanied by HPA axis activation, presumably because exogenous CCK activates hindbrain neurons that inhibit food intake and neurons that activate hypothalamic CRH neurons.

A2 neurons, including PrRP neurons, are necessary for the ability of a moderate to high dose of CCK to reduce meal size, and also are necessary for CCK-induced activation of neurons within the PVN (Rinaman, 2003a). Interestingly, DbH−/− mice (which cannot convert dopamine to NE) show no deficiencies in the ability of CCK to reduce food intake (Cannon and Palmiter, 2003), suggesting that PrRP rather than NE is the principal mediator of CCK-induced hypophagia, at least in mice. Indeed, a later study confirmed that PrRP signaling is necessary for the ability of exogenous CCK to suppress food intake in mice (Bechtold and Luckman, 2006). CCK activates cFos expression by GLP-1 neurons (Rinaman, 1999b; Maniscalco and Rinaman, 2013), but GLP-1 neuronal recruitment appears to be insufficient to support CCK hypophagia in rats with A2 neuronal loss, which would include loss of cNST PrRP neurons (Rinaman, 2003a). However, there are no published reports indicating whether GLP-1R signaling is necessary for CCK-induced hypophagia in either rats or mice.

### Lithium chloride

Peripheral administration of the nauseogenic agent LiCl, an experimental model of toxemia, potently increases plasma corticosterone and inhibits food intake in rats (McCann et al., 1989). Unlike satiation, LiCl reduces food intake in rats by reducing feeding frequency, without reducing meal size (West et al., 1987). While LiCl treatment activates cFos within A2 and GLP-1 neurons in rats and mice (Rinaman, 1999a,b; Lachey et al., 2005; Rinaman and Dzmura, 2007), one report indicates that a hypophagic dose of LiCl in rats does not activate the PrRP-positive subpopulation of A2 neurons (Lawrence et al., 2002). This result suggests that LiCl suppresses food intake through non-PrRP signaling pathways, and that only stimuli related to normal satiation are sufficient to recruit PrRP neurons, as hypothesized previously (Luckman and Lawrence, 2003). Pharmacological blockade of central GLP-1 receptors or selective lesions that destroy A2 neurons, likely including PrRP neurons, attenuates (but does not abolish) the ability of LiCl to inhibit food intake in rats (Rinaman, 1999a; Seeley et al., 2000; Kinzig et al., 2002; Rinaman and Dzmura, 2007), and central GLP-1R antagonism blunts LiCl-induced activation of the HPA axis (Kinzig et al., 2003). Central GLP-1R antagonism also decreases LiCl-induced cFos in the rat cNST (Thiele et al., 1998), evidence that GLP-1R signaling contributes to LiCl-induced recruitment of cNST neurons. Conversely, although LiCl activates GLP-1 neurons in mice, neither GLP-1R antagonism in wild type mice nor the absence of GLP-1R signaling in GLP-1R−/− mice attenuates the hypophagic effects of LiCl (Lachey et al., 2005). These disparate findings in rats and mice suggest important species differences in the role of GLP-1 signaling in responses to toxemia and other nauseogenic treatments.

### Immune challenge

Experimental models of infection, including systemic lipopolysaccharide (LPS; the major outer membrane component of gram-negative bacteria), promote the release of proinflammatory cytokines, elevate plasma levels of ACTH and corticosterone (Sapolsky et al., 1987; Hansen et al., 2000; Serrats and Sawchenko, 2006), and dose-dependently suppress food intake (Uehara et al., 1989; Kaneta and Kusnecov, 2005). Bacterial infections, LPS administration, and cytokines drive central stress responses via receptors located on vagal afferent terminals (Watkins et al., 1995; Fleshner et al., 1998; Goehler et al., 1999; Hosoi et al., 2005) and/or on endothelial and perivascular cells (Sawchenko et al., 2000; Zhang and Rivest, 2003), and signaling through both routes engages cNST neurons. LPS administered into the brain ventricles in rats reduces food intake primarily by reducing meal size (Plata-Salaman and Borkoski, 1993). Conversely, systemic administration of LPS reduces intake by reducing meal frequency (Langhans et al., 1990, 1993), suggesting that central and peripheral routes of administration engage different feeding control circuits. Systemic LPS increases hindbrain PrRP gene expression (Mera et al., 2006), and activates A2 neurons, GLP-1 neurons, and PVN neurons to express cFos (Rinaman, 1999b). PVN and HPA axis activation in response to immune challenge is significantly attenuated in rats after unilateral transection of ascending projections from the cNST to the PVN that interrupt both PrRP and GLP-1 signaling pathways (Li et al., 1996), or by selective neurochemical lesions of NA neurons (likely including PrRP neurons) that innervate the PVN (Bienkowski and Rinaman, 2008). Grill et al. (2004) demonstrated that LPS-induced hypophagia is dependent on hindbrain, but not forebrain, GLP-1R signaling. However, hypophagic (and HPA axis) responses to LPS are intact in GPR10−/− mice (Bechtold and Luckman, 2006), evidence that PrRP signaling is unnecessary for stress-induced hypophagia in this species. Taken together, these studies suggest that the hypophagic and HPA axis responses to bacterial infection and proinflammatory cytokines depend in large part on the recruitment of NA and GLP-1 neurons, while the role of PrRP signaling has been challenged in mice, and not yet explored in rats.

### Immobilization/restraint

One of the most commonly used models of cognitive stress is physical restraint, which activates the HPA axis and suppresses food intake (Rybkin et al., 1997; Kinzig et al., 2008; Seto et al., 2008; Calvez et al., 2011). A recent study investigated whether restraint and forced swim stress inhibited food intake in rats by reducing meal size, meal number, or both. Similar to satiation, restraint and forced swim stress both reduced food intake by reducing meal size and duration (Calvez et al., 2011), supporting the view that these stressors engage circuits that also are engaged by satiety signals. Restraint increases PrRP gene expression (Mera et al., 2006) and also activates cFos in NA neurons, including PrRP neurons (Dayas et al., 2001a,b; Maruyama et al., 2001; Banihashemi and Rinaman, 2011), apparently via descending projections from the PVN (Dayas and Day, 2001; Dayas et al., 2004). Recent findings in our laboratory indicate that restraint also activates GLP-1 neurons in rats (Maniscalco et al., 2012). The ability of restraint to inhibit food intake is closely linked to its ability to inhibit vagally mediated gastric emptying (Seto et al., 2008; Suzuki and Hibi, 2010). However, it currently is unknown whether the ability of restraint or any other cognitive stressor to decrease gastric emptying and food intake depends on central PrRP or GLP-1 receptor signaling.

## Conclusion

Stress affects both food intake and energy balance, and food intake can itself be stressful (Woods, 1991). For the purpose of this review, we set out to gather and interpret experimental evidence that satiety signals and stress engage a common set of neurons that contribute to the inhibition of food intake. Hindbrain PrRP and GLP-1 neurons satisfy many of the criteria that one might consider important for such a common set of neurons. Both neuronal populations are recruited to express cFos in animals exposed to satiety signals and many hypophagic stressors, and PrRP and GLP-1 signaling pathways impact body energy balance by reducing food intake and activating the HPA axis. Based on our review of the available literature, we propose that hindbrain PrRP and GLP-1 neurons represent important points of central integration in the control of energy intake and metabolism during feeding and in response to other acute homeostatic challenges. We do not argue that PrRP and GLP-1 neurons are the only important players in these coordinated processes. Instead, we present this evidence to establish a working hypothesis about the unique role played by these cNST neurons within the anatomically broad and complex neural systems that regulate energy homeostasis on a day-to-day basis. Experimental predictions arising from this hypothesis will be challenged by ongoing and future work in our laboratory.

## Conflict of Interest Statement

The authors declare that the research was conducted in the absence of any commercial or financial relationships that could be construed as a potential conflict of interest.

## Footnotes

^1^Amoeboid microglia also contain GLP-1 immunoreactive material, at least when activated in culture (Iwai et al., 2006), suggesting that under certain pathological conditions activated microglia may release GLP-1 to access receptors in brain sites not normally targeted by the axons of hindbrain GLP-1 neurons.

^2^HPA axis recruitment by PrRP and GLP-1 signaling pathways is discussed further in the Section “Potential Role of PrRP and GLP-1 Neurons in Stress-Induced Hypophagia” of this review.
